# Getting small to feel big: the psychology of weight cutting in combat sports

**DOI:** 10.3389/fspor.2025.1495612

**Published:** 2025-01-22

**Authors:** Jacob J. Levy, Christopher Boyd

**Affiliations:** Department of Psychology, University of Tennessee, Knoxville, TN, United States

**Keywords:** rapid weight loss, martial arts, exercise motivation, body image, performance psychology

## Abstract

This mini review explores the psychological factors associated with weight cutting practices among combat sport athletes. We overviewed combat sport athletes' extrinsic and intrinsic motivations for their sport participation and performance expectations and goals associated with weight cutting. Next, we reviewed the extant research on psychological risk factors associated with weight cutting with a particular focus on combat sport athletes' relationship with food, societal expectations concerning body image, and disordered eating. Finally, we examined how applying task-oriented strategies and Goal Attainment Theory aligns with combat sport athletes' motivation for sport participation. We called for further research into exploring how promoting gradual weight loss may promote psychological resilience with the goal of promoting healthier weight management strategies among combat sport athletes.

## Introduction

In combat sports, athletes often compete against opponents with shared gender, skill level, and weight class. A common practice among combat sport athletes is *weight cutting* or losing body weight in the weeks and often days leading up to the competition. Previous investigations have found 60%–80% of combat sport athletes have reported engaging in some form of weight cutting prior to competition (1), with the highest prevalence of weight cutting being found among boxers and mixed martial arts (MMA) athletes (2). Boxing and MMA typically perform weigh-ins 24–36 h prior to competition, thus allowing for some time to partially rehydrate prior to their contest. Many of these athletes engage in rapid weight loss (RWL)–losing at least 5% of their body weight, typically through drastic dehydration methods, in the days or hours leading up to the competition weigh-in (3). During this recovery time following weigh-in, many of these athletes will gain back most of the body mass lost during their weight before entering their contest*—*referred to as rapid weight gain (RWG). Research on the physical health risks associated with weight cutting have found that athletes who engage in RWL/RWG practices are more prone to in-competition injury and other, potentially life threatening, health problems including cardiovascular problems, stroke, concussion proneness, heat illness (including heat stroke), hormonal imbalance, and changes in insulin sensitivity [c.f., (1, 4)]. The rationale combat sport athletes often give for engaging in weight cutting practices is to gain a competitive advantage on their opponent by competing against smaller or lighter opponents (1, 5). However, a recent meta-analysis on the effects of weight cutting on performance outcomes among combat athletes found no substantial benefit or negative effects relative to performance success (6). Thus, there appears to be a disconnect between the athlete's perception of the performance advantages of weight cutting vs. the research findings suggesting, at best, no substantial performance benefit and, at worst, potential serious health consequences. *So why do they do it?* The purpose of this mini review is to explore the psychological factors associated with weight cutting practices among combat sport athletes. Specifically, we review the motivations and goals for engaging in weight cutting, psychological risks associated with RWL/RWG and offer our perspective regarding future directions for research into promoting a more psychologically and physically healthy approaches to weight management in combat sport.

## Motivations for weight cutting

Combat sport athletes often view weight management as an essential part of their competitive strategy. For example, a combat sport athlete would rather be at the top of a lighter weight class, than the bottom of a heavier weight class (5). In terms of external sources of motivation for this practice, combat sports culture often glorifies extreme weight-cutting practices, viewing RWL as a symbol of discipline and dedication (7). These cultural norms begin early in a combat athletes' training—often in childhood—and persist throughout their athletic career (36). RWL practices are often encouraged by coaches and fellow athletes, rather than by qualified health professionals like physicians and dietitians (7). Coaches and peers, who may lack formal training in nutrition or health, frequently advocate for RWL based on anecdotal evidence rather than scientific knowledge. This reliance on non-expert advice can lead athletes to engage in RWL without fully considering potential health risks, from acute to chronic complications (8). Thus, the continuation of unhealthy practices may be associated, in part, to the desire to please or be praised by coaches and peers.

From an intrinsic motivation perspective, research suggests that combat sport athletes are often drawn to and remain in their sport because their intrinsic need for challenge, accomplishment, and competence (5, 9, 10). For female combat sport athletes, Mathisen and colleagues (11) found the desire to gain strength, learn self-defense, and feel more confident often drives women to combat sports, which are traditionally male-dominated, and challenge societal expectations of female behavior. They also highlight the inclusive and supportive community within combat sports, which fosters feelings of belonging, further contributing to empowerment. The sense of control that comes with participating in combat sports may be linked to the prevalence of weight regulation practices, as female athletes often seek to manage their bodies to meet weight class requirements and optimize performance. For both male and female combat athletes, successfully reaching weight targets can enhance motivation, self-esteem, and a sense of accomplishment, fostering positive mental health and psychological resilience (12, 13). However, failure to meet weight goals or the absence of visible progress can lead to self-doubt, diminished mental readiness, and heightened anxiety (13).

*Horrible–but worth it* (5) is a statement combat sport athletes often offer when describing the RWL/RWG practices. Engaging in this challenging practice may be meeting their intrinsic motivational needs, as well being externally reinforced by their coach and peers. However, we posit weight cutting may also provide a built-in excuse for performance failure, which may serve the function of minimizing threats to self and self-worth. For example, a loss may be attributed to a *bad weight cut* as opposed to those that may be more personal (e.g., the other fighter was more skilled). Further research is needed to explore and clarify the relationship among these intrinsic and extrinsic motives for weight cutting practices.

## Weight cutting, body image, and cultural norms

In additional the physical health risks associated with weight cutting practices, the process of engaging in RWL/RWG can also impact the combat sport athlete's relationship with food, disordered eating, and are linked to negative psychological outcomes including increased stress, nervousness, tension, fatigue, and anger (14, 15). Disordered eating (DE) is prevalent among combat athletes, with 83% of males and 89% of females exhibiting moderate to very high DE scores post-competition (16). The mental strain caused by weight management pressures, particularly those linked to RWL, contributes to struggles with food control, bingeing, and body dissatisfaction. The link between restrictive eating and emotional eating is another challenge for athletes. Restrained eating, common among combat athletes, can increase the likelihood of emotional eating, particularly after competitions. Barker and colleagues (17) identified that efforts to maintain dietary restraint can deplete self-control, making it harder to resist emotional eating. According to the limited capacity theory, individuals have a finite number of cognitive resources for self-control, and when these resources are depleted by the effort of regulating emotions, their ability to manage food intake may be compromised. Self-control theory posits that engaging in one self-control task, such as dietary restraint, can hinder self-control in other areas, like resisting emotional eating, especially when individuals face multiple self-control demands or feel depleted. Athletes reported a cycle of restrictive eating before competition followed by emotional eating after, particularly if they lost. This behavior aligns with theories like the boundary model of eating, which explains that restrained eaters often set strict dietary rules for themselves. When they perceive a violation of these rules, they may experience the *what-the-hell effect*, leading to overeating (17). Interventions such as emotional eating diaries, mindful eating, and alternative coping strategies can help athletes manage these challenges (17). Interestingly, while male athletes often experience declining body satisfaction post-competition, female athletes show improvements, suggesting a potential sex-specific temporal relationship with DE (16). Regardless of sex, the severity of negative mood states appears to be directly proportional to the magnitude of weight loss within a given timeframe, indicating that more aggressive RWL methods could have greater negative impacts on athletes' mental well-being (37).

Despite the psychological risks, some athletes continue to use weight cutting to gain a competitive psychological advantage, believing that appearing leaner or lighter projects strength (13). However, the long-term emotional and psychological effects of these practices require interventions to promote healthier relationships with food and body image (16). Cultural norms and media portrayals strongly influence body image and weight management in athletes. Media-driven ideals of body types also contribute to athletes' concerns about muscularity and lead to unhealthy weight management behaviors (13, 18). For example, in collegiate wrestling, extreme weight management practices are deeply embedded in the sport's culture. This normalization reinforces the belief that weight cutting is an integral aspect of the sport, despite its physical and psychological toll (12). This normalization exacerbates body dissatisfaction and fosters unhealthy eating patterns (16), as athletes conform to the pressures of weight-class sports. Research shows that many judo athletes first engage in RWL as young as four years old, with more common onset around ages nine to twelve (19–21). This suggests that RWL/RWG is a heritage younger combat sport athletes inherit from older counterparts, who already experience the adverse effects of RWL, such as distorted self-image, negative mood profiles, impaired short-term memory, high susceptibility to eating disorders, and menstrual dysfunction (22). Coaches are identified as the primary source of information regarding RWL methods, with few athletes consulting health professionals for weight management advice. This suggests that RWL practices are passed down through tradition rather than based on evidence-based or scientifically sound approaches (23).

Fasczewski and colleagues (24) highlight male athletes, especially in weight-class sports, face intense pressure to conform to ideals of muscularity, often leading to extreme dieting and excessive exercise. This drive is reinforced by cultural norms that equate muscularity with strength and dominance, particularly in combat sports. Satterfield and Stutts (38) observe that male wrestlers frequently engage in severe weight-cutting practices to meet weight class requirements while enhancing muscle mass, reflecting the broader cultural emphasis on size and strength as markers of athletic success.

Research shows that female combat sport athletes face unique societal pressures, with higher levels of disordered eating, such as restrictive dieting and excessive exercise, compared to male athletes (16). These pressures, combined with weight-cutting practices, increase the risk of developing conditions associated with the female athlete triad—disordered eating, menstrual dysfunction, and low bone mineral density (25, 26). Prolonged exposure to these conditions can significantly harm athletes' health and shorten their competitive careers (27, 28). Moreover, the tension between maintaining a lean, feminine body ideal and the muscular physique required for combat sports leads to heightened body dissatisfaction and extreme weight management practices (29, 30).

## Gradual weight loss approaches and psychological resilience

Combat sports often normalize RWL practices driven by the immediate pursuit of competitive advantages. However, evidence indicates that a gradual approach—defined as 0.5–1 kg per week—not only aligns better with athletes' long-term health and performance goals but also supports psychological well-being (31). Gradual weight loss preserves lean body mass and enhances performance compared to rapid weight loss methods, which can lead to detrimental effects on body composition and strength (8). Athletes who adhere to a slower rate of weight loss experience improvements in lean body mass, strength, and power-related performance, which are critical for sustaining high-level performance in combat sports. Fogelholm and colleagues (31) further demonstrate that gradual weight loss results in better muscle mechanical functioning, such as increased vertical jump height, compared to rapid methods. Additionally, Miranda and colleagues (39) highlight that gradual weight loss contributes to better maintenance of performance metrics and reduces the adverse effects often observed with rapid weight loss methods.

From a psychological perspective, resilient athletes tend to use task-oriented coping strategies, focusing on practical and proactive ways to manage challenges (32). Crust and Clough provide a clear connection between mental toughness, gradual goal-setting, and long-term resilience. By encouraging slow, deliberate weight loss, athletes can build the mental toughness necessary to handle the physical and psychological demands of competition while maintaining their well-being. Crust and Clough (32) argue that achieving long-term goals, which require significant time and dedication, can enhance self-belief and perceptions of competence—aligning with their intrinsic motivations for sport participation. The process of working towards these goals helps athletes develop resilience and mental toughness.

Gradual weight loss aligns with Goal Attainment Theory by breaking down large, long-term goals into smaller, manageable steps. This approach fosters resilience by allowing athletes to focus on incremental progress rather than short-term fixes. Resilient athletes are better at managing stress and anxiety, which are significant during weight management (33). Gradual weight loss supports task-oriented coping strategies, which focus on improving specific aspects of performance and managing stress in a controlled manner. By setting daily or weekly goals, athletes can maintain a sense of control and manage pressure effectively, reducing the overwhelming stress associated with rapid weight loss. Conversely, rapid weight loss can overwhelm athletes, leading to negative appraisals and increased anxiety, which adversely affects performance (34). Coaches and athletes should be encouraged to adopt gradual weight loss strategies to promote healthier mental states and prevent the negative psychological outcomes associated with rapid weight loss.

## Conclusion and future directions

Combat sports culture often glorifies extreme weight-cutting practices (7). This cultural norm, deeply ingrained in the sport, can overshadow the benefits of a more controlled and gradual approach to weight management. While weight regulation is culturally significant, it's essential to balance these norms with health considerations. Gradual weight loss can be framed as both a culturally acceptable and health-conscious strategy, allowing athletes to meet cultural expectations while preserving their well-being. By integrating gradual weight loss into the cultural narrative, coaches can demonstrate that maintaining health is compatible with achieving sport-specific goals, thus promoting a more sustainable and health-focused approach. This shift can enhance athletes' self-image, reduce stress, and support overall psychological resilience and health (31, 35).

Educating athletes, coaches, and support staff about the benefits of gradual weight loss and the psychological advantages associated with task-oriented coping strategies may facilitate a shift in practices and perspectives. Providing knowledge about the long-term benefits of gradual weight loss and encouraging the use of effective coping strategies—such as problem-solving and goal setting—may help athletes manage the challenges of weight cutting more effectively. Implementing gradual weight loss strategies in combat sports may offer numerous advantages, including better preservation of lean body mass, enhanced performance, and improved psychological resilience. Additional research is needed to the explore the shifting cultural norms and promoting education about the benefits of gradual weight loss to foster a more supportive and effective training environment. Such a future focus may not only benefits athletes' physical health but also may also support their mental well-being, ultimately contributing to sustained success in combat sports.
